# IL-19 Contributes to the Development of Nonalcoholic Steatohepatitis by Altering Lipid Metabolism

**DOI:** 10.3390/cells10123513

**Published:** 2021-12-13

**Authors:** Yasu-Taka Azuma, Takashi Fujita, Takeshi Izawa, Kana Hirota, Kazuhiro Nishiyama, Airi Ikegami, Tomoko Aoyama, Mikihito Ike, Yumi Ushikai, Mitsuru Kuwamura, Hideki Fujii, Koichi Tsuneyama

**Affiliations:** 1 Laboratory of Veterinary Pharmacology, Division of Veterinary Science, Osaka Prefecture University Graduate School of Life and Environmental Sciences, Osaka 598-8531, Japan; hirota@vet.osakafu-u.ac.jp (K.H.); knishiyama@phar.kyushu-u.ac.jp (K.N.); ushikai@vet.osakafu-u.ac.jp (Y.U.); 2 Molecular Toxicology Laboratory, Department of Pharmaceutical Sciences, Ritsumeikan University, Kusatsu 525-8577, Japan; fujitat@fc.ritsumei.ac.jp (T.F.); aikegami@ed.ritsumei.ac.jp (A.I.); taoyama@ed.ritsumei.ac.jp (T.A.); mike@ed.ritsumei.ac.jp (M.I.); 3 Laboratory of Veterinary Pathology, Division of Veterinary Science, Osaka Prefecture University Graduate School of Life and Environmental Sciences, Osaka 598-8531, Japan; izawa@vet.osakafu-u.ac.jp (T.I.); kuwamura@vet.osakafu-u.ac.jp (M.K.); 4 Department of Premier Preventive Medicine, Graduate School of Medicine, Osaka City University, Osaka 545-8585, Japan; fujiirola@yahoo.co.jp; 5 Department of Pathology and Laboratory Medicine, Institute of Biomedical Sciences, Tokushima University Graduate School, Tokushima 770-8503, Japan; koichi.tsuneyama@gmail.com

**Keywords:** IL-19, NAFLD, NASH, inflammation, liver, lipogenesis

## Abstract

Interleukin (IL)-19, a member of the IL-10 family, is an anti-inflammatory cytokine produced primarily by macrophages. Nonalcoholic steatohepatitis (NASH) is a disease that has progressed from nonalcoholic fatty liver disease (NAFLD) and is characterized by inflammation and fibrosis. We evaluated the functions of IL-19 in a NAFLD/NASH mouse model using a 60% high fat diet with 0.1% methionine, without choline, and with 2% cholesterol (CDAHFD). Wild-type (WT) and IL-19 gene-deficient (KO) mice were fed a CDAHFD or standard diet for 9 weeks. Liver injury, inflammation, and fibrosis induced by CDAHFD were significantly worse in IL-19 KO mice than in WT mice. IL-6, TNF-α, and TGF-β were significantly higher in IL-19 KO mice than in WT mice. As a mechanism using an in vitro experiment, palmitate-induced triglyceride and cholesterol contents were decreased by the addition of IL-19 in HepG2 cells. Furthermore, addition of IL-19 decreased the expression of fatty acid synthesis-related enzymes and increased ATP content in HepG2 cells. The action of IL-19 in vitro suppressed lipid metabolism. In conclusion, IL-19 may play an important role in the development of steatosis and fibrosis by directly regulating liver metabolism and may be a potential target for the treatment of liver diseases.

## 1. Introduction

Interleukin (IL)-19 is a member of the IL-10 family and is produced primarily by macrophages [1]. We have previously analyzed the role of IL-19 in inflammatory bowel disease and dermatitis. In disease model mice of Crohn’s disease [2], ulcerative colitis [3,4], and contact hypersensitivity [5,6], we found that IL-19 gene-deficient (KO) mice showed an exacerbation of symptoms, and IL-19 acted as an inhibitor of colon and cutaneous inflammation. In addition to these reports, there are reports on the role of IL-19 in asthma, the central nervous system, and joints [7]. However, the role of IL-19 in the liver, including liver inflammation and chronic liver disease, has not been reported at all.

Nonalcoholic fatty liver disease (NAFLD) is a liver disorder that resembles alcoholic liver disease, despite the absence of an obvious drinking history [8]. The most important factor involved in the development of NAFLD is obesity [8]. The accumulation of triglycerides (TGs) leads to the development of a nonalcoholic fatty liver (NAFL). Nonalcoholic steatohepatitis (NASH) is an advanced form of NAFL in which there is an infiltration of inflammatory cells and fibrosis in liver tissue similar to alcoholic steatohepatitis [9]. NAFLD is a disease that includes NAFL and NASH. In epidemiology, NAFLD is associated with dyslipidemia, hypertension, fasting hyperglycemia, and metabolic syndrome [10,11]. NASH is more strongly associated with the above four diseases than NAFLD. In terms of the progression of inflammatory cell infiltration and fibrosis, it is basically believed that inflammatory cell infiltration occurs first, followed by fibrosis. Therefore, we wondered if IL-19, which is involved in inflammation in inflammatory bowel disease and dermatitis, might play some role in liver inflammation in NASH progression. Furthermore, there are no reports of IL-19 and fibrosis in other organs, as well as in the liver. There are several reports on NAFLD/NASH of IL-10 family members other than IL-19, such as IL-20, IL-22, and IL-24 [12]. Therefore, we determined that the analysis of the role of IL-19 in NASH is an objective of high research value.

There are two ways to study NAFLD/NASH: using animals that develop NAFLD/NASH and diets that cause NAFLD/NASH. A number of mouse models with special diets have been reported so far. In the first period, diet without methionine and choline was used [13], followed by a 60% high fat diet [14]. The next diet was a 60% high fat diet without methionine and choline [15]. However, these diets did not replicate the human NASH phenotype, although they showed histological evidence of hepatocellular steatosis. Subsequently, modifications are made to the diet composition and the latest diet is a 60% high fat diet with 0.1% methionine without choline [16]. This improved diet progressed rapid hepatic fibrosis but required 12 weeks for clear fibrosis. In this study, we focused on cholesterol [17] and used a 60% high fat diet with 0.1% methionine, without choline, and with 2% cholesterol for 9 weeks, believing that a shorter duration would be useful in developing an efficient treatment. We investigated the novel role of IL-19 in a diet-induced NAFLD/NASH model using IL-19 KO mice. We firstly observed that a diet-induced steatosis and fibrosis was exacerbated in IL-19 KO mice. In addition, we conducted an analysis of the mechanism of IL-19 in vitro and showed that IL-19 inhibited lipid metabolism, especially the biosynthesis of TGs.

## 2. Materials and Methods

### 2.1. Mice

We used IL-19 KO mice on a C57BL/6 background that we previously generated [4]. Heterozygous mice were crossed to produce IL-19 KO and wild-type (WT) as control mice. All mice used were 7–8-week-old males for NAFLD/NASH mouse model experiments and 10-week-old males for in vitro experiments. All animal protocols were approved by the Osaka Prefecture University Animal Care and Committee. All procedures used in this study complied with institutional policies of the Osaka Prefecture University Animal Care and Use Committee.

### 2.2. NASH Induction

The diet (A06071315) fed in this study was 60% high fat, 0.1% methionine, and 2% cholesterol without choline (CDAHFD), and were purchased from Research Diets (New Brunswick, NJ, USA). Male mice were fed a CDAHFD diet for 9 weeks. After 9 weeks, all mice were euthanized under anesthesia with isoflurane. The liver, spleen, and kidneys were taken, and the weight of each was recorded. Blood samples were collected from the heart and centrifuged at 10,000× *g* for 10 min.

### 2.3. Kupffer Cells, Hepatocytes Isolation and Immunocytochemical Analysis

Kupffer cells and hepatocytes were prepared from liver. Briefly, a 19G puncture needle was cannulated into the inferior vena cava of a euthanized 10-week-old mouse, and the superior vena cava was clipped with a disposable clip (AS-1-20) (Natsume Seisakusho Co., Ltd., Tokyo, Japan). Then, HBSS (−) + 0.005 M EDTA was gradually refluxed into the inferior vena cava, and the distal part of the barrier vein was immediately incised to remove blood. Five milliliters of HBSS (−) + 0.005 M EDTA was gently refluxed, followed by 5 mL of HBSS (−). After further clipping the hepatic portal vein, the 0.025 mg/mL collagenase (FUJIFILM Wako Pure Chemical Corporation, Osaka, Japan) in 5 mL of serum-free RPMI1640 was refluxed. The operation up to this point was maintained at 4 °C. Then, the shredded liver in a C tube was reacted for 30 min using a Gentle MACS (Miltenyi Biotech, Bergisch Gladbach, Germany) hepatocyte preparation program. After reaction, HBSS (−) was added to the reaction solution and was filtered through a cell strainer with 70 μm nylon mesh (BD falcon, NJ, USA). Cell suspensions were centrifuged at 70× *g* for 3 min. The first supernatant was gently transferred to another tube (including Kupffer cells and sinusoidal endothelial cells). The pellet containing the hepatocytes were gently washed 8 times with HBSS (−) and then seeded on collagen-coated plates at a concentration of 1–3 × 10^5^ cells/mL. A 10% FCS DMEM/F12 medium supplemented with 50 ng/mL corticosterone, 50 ng/mL triiodothyronine, and 10 ng/mL EGF for hepatocytes, and a 10% FCS RPMI1640 medium for Kupffer cells, were used. Isolated Kupffer cells were purified by adhesion to a plastic plate and cultured in an 8-well chamber slide (Millicell^®^ EZ SLIDE) (Merck Millipore Ltd., Carrigtwohill, Ireland) for 3 days, then cultured in the presence of 2 μM monensin for 4 h before staining. Kupffer cells were plated to the slide.

Kupffer cells were fixed by 10% (*w/v*) neutral buffered formalin solution. Cells were stained with anti-F4/80 PE-conjugated (1:500) (53-4801-80, Thermo Fisher Scientific, Waltham, MA, USA), anti-IL-19 (1:500) (ab198925, Abcam, Cambridge, UK), anti-rabbit IgG Alexa Fluor™488 conjugated, and DAPI (4′,6-diamidino-2-phenylindole dihydrochloride). All antibodies were used at a 1:500 dilution. For the staining of liver paraffin sections, HistoVT One antigen retrieval solution (Nacalai Tesque, Kyoto, Japan) was used. Fluorescence images were obtained using microscopy (BX51/DP74) and cellSens software (Olympus, Tokyo, Japan).

### 2.4. In Vitro Steatosis Assay in HepG2 Cells

HepG2 cells were purchased from Riken Cell BANK (Ibaraki, Japan) and cultured in DMEM supplemented with 10% FBS and antibiotics. Palmitate-BSA was prepared according to the method of Joshi-Barve et al. [18]. Briefly, palmitate (Sigma Chemicals, Saint Louis, MI, USA) was mixed with 10% fatty acid-free BSA for 1-day at 37 °C and yielded 8 mM palmitate-BSA. Palmitate-BSA was treated to HepG2 cells for 48 h in the presence or absence of IL-19 (PeproTech, Cranbury, NJ, USA).

The cells were prepared separately and lysed by lysis buffer (10 mM Tris, pH 7.4, 1 mM EDTA, 0.1% Triton X-100), and respective markers were analyzed using Triglyceride-Glo™ Assay, Cholesterol/Cholesterol Ester-Glo™ Assay and ENLITEN ATP Assay System Bioluminescence Detection Kit (Promega, Madison, WI, USA).

The cells were stained with 1 µg/mL BODIPY™ 493/503 (4,4-Difluoro-1,3,5,7,8-Pentamethyl-4-Bora-3a,4a-Diaza-s-Indacene) (Thermo Fisher Scientific, Waltham, MA, USA). Cells were fixed and mounted using ProLong™ Gold Antifade Mountant with DAPI (Thermo Fisher Scientific, Waltham, MA, USA), and fluorescence images were obtained using the EVOS^®^ FL cell imaging system (Thermo Fisher Scientific, Waltham, MA, USA).

### 2.5. Reporter Cells

The neomycin cassette cloned by PCR was incorporated into NdeI sites of peroxisome proliferator-activated receptor (PPAR)-response element (PPRE)×3-TK-luc (addgene: #1015) and generated PPRE-luc-neo. pGL4.47 [luc2P/sis-inducible element (SIE)/Hygro] vector, purchased from Promega. Stably overexpressed HepG2 cells were generated by the Gene Pulser Xcell Electroporation System (Bio-Rad Laboratories Inc., Hercules, CA, USA). A total of 300 µg of PPRE-luc-neo or pGL4.47 and 2 × 10^7^ cells in PBS were transferred to a 4-mm gap electroporation cuvette, and electroporation was done at 200 V with a 1.5 ms pulse length. Clones were selected by 0.8–2 mg/mL G418 or 100 µg/mL hygromycin and isolated independently. Reporter activities were evaluated using a One-Glo Luciferase Assay System (Promega). Luminescence activities were measured using a model GloMax^®^ Discover Microplate Reader (Promega).

### 2.6. Aminotransferase and Lactose Dehydrogenase Levels

Activities of alanine aminotransferase (ALT) and aspartate aminotransferase (AST) in the serum and supernatants of cultured cells were immediately measured using a Transaminase CII test WAKO (Wako Pure Chemical, Osaka, Japan), as previously described [19]. Lactose dehydrogenase (LDH) release was examined using LDH-Glo™ Cytotoxicity Assay (Promega).

### 2.7. Liver Histology and Immunohistochemical Analysis

The liver was fixed with 10% neutral buffered formalin and embedded in paraffin blocks. Hematoxylin and eosin (H&E) staining was performed, as previously described [20]. Azan staining was performed to assess hepatic fibrosis. CD68 staining was performed to assess macrophage infiltration, as previously described [21]. The immunoreactivity of CD68 was detected using the DAB system. Images were captured using a VS120 Virtual Slide Microscope (Olympus Corporation, Tokyo, Japan). Liver histology was assessed by quantification using Image J.

### 2.8. RNA Isolation and Quantitative Real-Time PCR (QPCR)

Isolated liver tissues or cells were homogenated. Total RNA was isolated using Sepasol (Nacalai Tesque, Kyoto, Japan), and isolated RNA was used to synthesize complementary cDNA using SuperScript Reverse Transcriptase (Roche, Madison, WI, USA), as previously described [22]. Quantitative real-time PCR analysis based on the intercalation of SYBR Green (Toyobo, Osaka, Japan) were performed, as described previously [23]. Primer sequences used are summarized in Supplementary Table S1. A non-regulated housekeeping gene HPRT or GAPDH served as an internal control and was used to normalize for differences in input RNA.

### 2.9. Western Blot

Western blot analysis was performed, as described previously [24]. Briefly, 50 µg total proteins were blotted to 10% SDS-PAGE and transferred to PVDF membrane and reacted with anti-phosphorylated-STAT3 (1:1000 dilution) or phosphorylated-AMPK (1:1000 dilution) (Cell Signaling Tech., Danvers, MA, USA) and anti-Actin (1:1000 dilution) (Santa Cruz Biotechnology, Inc., Santa Cruz, CA, USA) and then exposed to horseradish peroxidase-conjugated anti-rabbit or anti-goat IgG (Santa Cruz Biotechnology, Inc., Santa Cruz, CA, USA). Chemiluminescence signals were obtained from reaction with Chemi Lumi One Plus Reagent (Nacalai Tesque), and data were obtained using an LAS4000 system (FUJI film, Tokyo, Japan).

### 2.10. Statistical Analysis

Liver weight, ALT/AST quantification, QPCR, TG and cholesterol contents, PPRE-luciferase and SIE-luciferase activities, and ATP and LDH contents were analyzed with one-way ANOVA for non-repeated measures to detect differences among groups. The differences between groups were determined using the Tukey–Kramer test. Other data were evaluated using the two-tailed Student’s *t*-test (unpaired) to detect differences between 2 groups. A *p* value of less than 0.05 was considered statistically significant.

## 3. Results

### 3.1. IL-19 Expression in the Kupffer Cells

**Figure 1:**
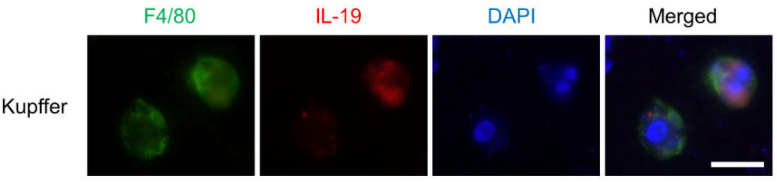
IL-19 expression in the Kupffer cells. Primary Kupffer cells were fixed, then reacted with IL-19 and F4/80 antibodies with DAPI. Representative images were shown (*n* = 3). Scale bar represents 50 μm.

We examined the IL-19 expression in the Kupffer cells isolated from WT mice because previous reports have shown that IL-19 is expressed in macrophages [1] and microglia [25]. As shown in Figure 1, we show that F4/80^high^ Kupffer cells in the liver expressed IL-19.

### 3.2. Body and Liver Weights

**Figure 2:**
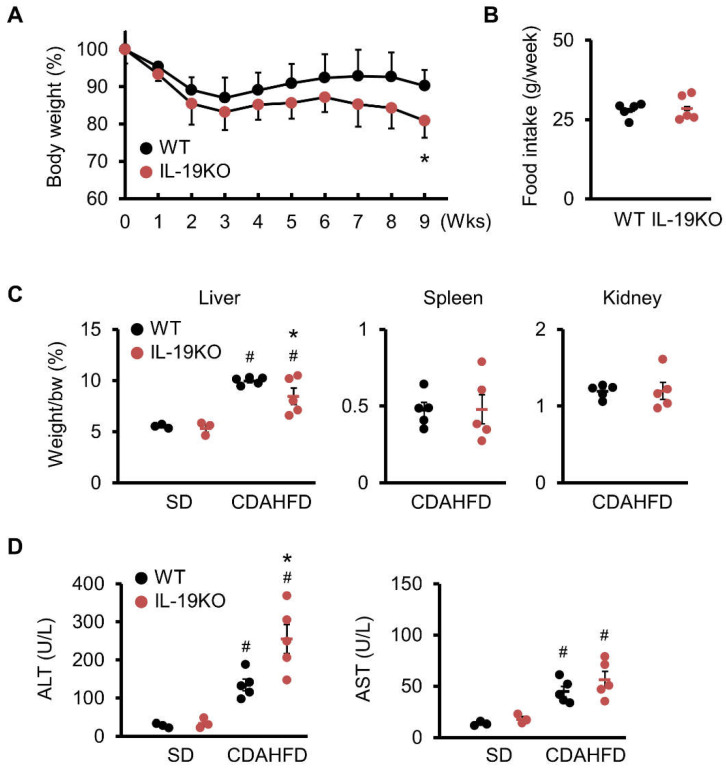
The changes in body weight, food intake, organ weights, and serum ALT and AST levels. WT (*n* = 15) and IL-19 KO (*n* = 15) mice were fed a CDAHFD diet for 9 weeks. Body weight (*n* = 15) (**A**) and food intake (*n* = 5) (**B**) were monitored weekly. (**C**) Liver weight, spleen weight, and kidney weight were determined at week 9 (*n* = 5). Liver weight was also measured in the SD-fed group (*n* = 3). (**D**) The ALT and AST levels in the serum were measured (*n* = 5). The ALT and AST levels in the SD group was also measured as a control group (*n* = 3). * *p* < 0.05 vs. WT. ^#^ *p* < 0.05 vs. SD.

We next examined the body weights of mice fed a CDAHFD diet for 9 weeks. WT mice lost body weight until week 4 but then recovered. The body weight of IL-19 KO mice decreased in the same manner as WT mice up to week 4 but then recovered more slowly than WT mice. After week 8, the body weight difference between IL-19 KO and WT mice increased, and the body weight of IL-19 KO mice was significantly reduced at week 9 (Figure 2A). We confirmed that the food intake in IL-19 KO mice was similar to that in WT mice (Figure 2B). Both WT and IL-19 KO mice fed the CDAHFD diet had significantly increased liver weight than those fed the standard diet (SD) (Figure 2C). Importantly, the liver weight of IL-19 KO mice was significantly reduced at week 9 (Figure 2C). In contrast, both spleen and kidney weights were similar in WT and IL-19 KO mice (Figure 2C).

### 3.3. ALT and AST

Blood analysis of liver profiles showed that levels of liver-damaging enzymes, such as ALT and AST, were significantly increased in WT and IL-19 KO mice fed the CDAHFD diet compared to those fed the SD diet (Figure 2D). Only ALT was significantly increased in IL-19 KO mice compared to WT mice at week 9.

### 3.4. Liver Histology

**Figure 3:**
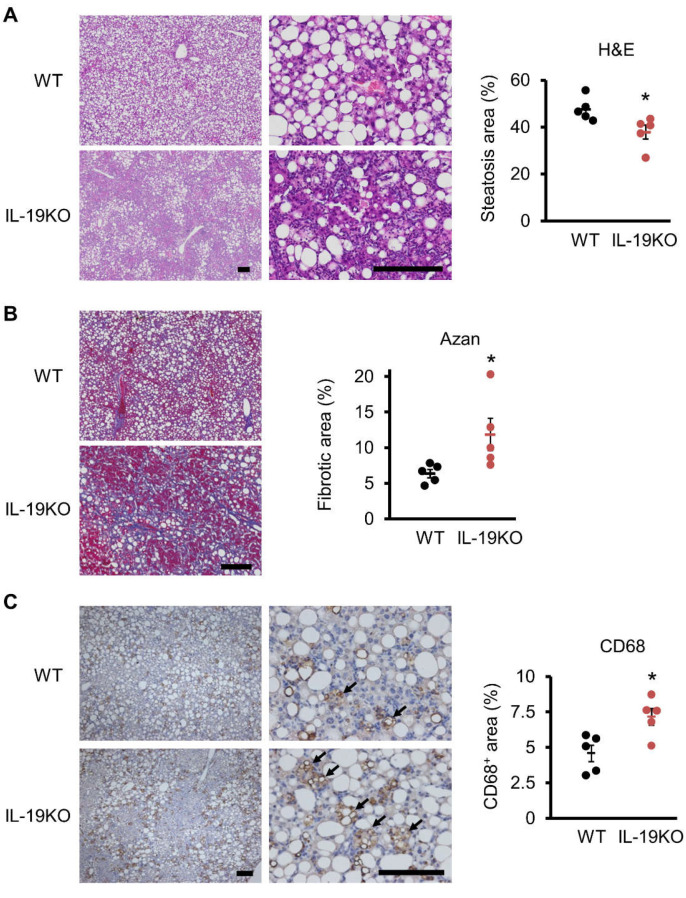
H&E, Azan, and CD68 staining. WT and IL-19 KO mice were fed a CDAHFD diet for 9 weeks. (**A**) Representative liver sections stained with H&E are shown. Low and high magnification are shown. Empty envelopes were quantified using ImageJ and evaluated as steatosis (*n* = 5). (**B**) Representative liver sections stained with Azan staining are shown. The blue color was quantified using ImageJ and evaluated as fibrosis (*n* = 5). (**C**) Representative liver sections immunohistochemically stained with CD68 are shown. Low and high magnification are shown. Positive cells were quantified using ImageJ (*n* = 5). Scale bars represent 200 μm. * *p* < 0.05 vs. WT.

Histopathology data showed that WT mice fed a CDAHFD diet caused predominantly middle droplet steatosis and induced infiltration of inflammatory cells, as stained with H&E (Figure 3A). Fibrosis is less noticeable in WT mice, as stained with Azan staining (Figure 3B). In IL-19 KO mice fed a CDAHFD diet, the size of droplets was intermediate, similar to WT mice, but steatosis was significantly weaker than WT mice (Figure 3A). In addition, fibrosis was significantly stronger in IL-19 KO mice than in WT mice (Figure 3B). Infiltration of inflammatory cells was markedly higher in IL-19 KO mice than in WT mice. Infiltration of inflammatory cells was quantified by immunostaining with CD68, a macrophage marker. There were significantly more CD68-positive cells in IL-19 KO mice than in WT mice (Figure 3C).

### 3.5. IL-19 Expression and Factors Involved in NASH Progression

**Figure 4:**
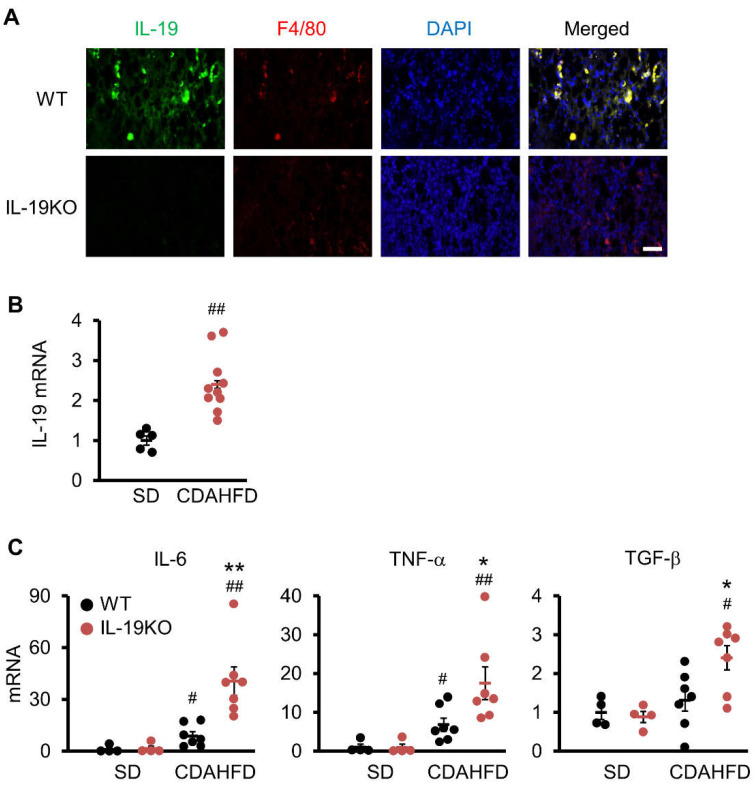
IL-19 expression in the liver and quantitative real-time PCR. WT and IL-19 KO mice were fed a CDAHFD diet for 9 weeks. (**A**) Representative liver sections immunohistochemically stained with IL-19 and F4/80 antibodies with DAPI are shown (*n* = 4–5). Scale bar represents 50 μm. (**B**) IL-19 mRNA expression in the livers of WT mice fed an SD (*n* = 5) or CDAHFD (*n* = 10) diet for 9 weeks. ^##^ *p* < 0.01 vs. SD. (**C**) mRNA expressions of IL-6, TNF-α, and TGF-β in the livers of WT and IL-19 KO mice fed an SD (*n* = 4) or CDAHFD (*n* = 7) diet for 9 weeks. ^#^ *p* < 0.05, ^##^ *p* < 0.01 vs. SD. * *p* < 0.05, ** *p* < 0.01 vs. WT.

We analyzed the expression of IL-19 in WT mice fed a CDAHFD diet for 9 weeks. Immunostaining of liver tissues showed that IL-19 in F4/80^high^ Kupffer cells was clearly expressed, and the specificity of the antibody was also confirmed, as IL-19 signal was not detected in IL-19 KO mice (Figure 4A). The QPCR results showed that feeding CDAHFD significantly increased the expression of IL-19 in WT mice compared to SD (Figure 4B).

IL-6 and TNF-α were measured as factors involved in inflammation. Both factors were significantly increased in WT and IL-19 KO mice fed the CDAHFD diet compared to those fed the SD diet. In addition, both factors were significantly increased in IL-19 KO mice compared to WT mice (Figure 4C). TGF-β were measured as an important factor involved in fibrosis. TGF-β was significantly increased in IL-19 KO mice, but not WT mice, for those fed the CDAHFD diet compared to those fed the SD diet. In addition, TGF-β was significantly increased in IL-19 KO mice compared to WT mice (Figure 4C).

### 3.6. Effect of IL-19 on In Vitro Steatosis Model in HepG2 Cells

**Figure 5:**
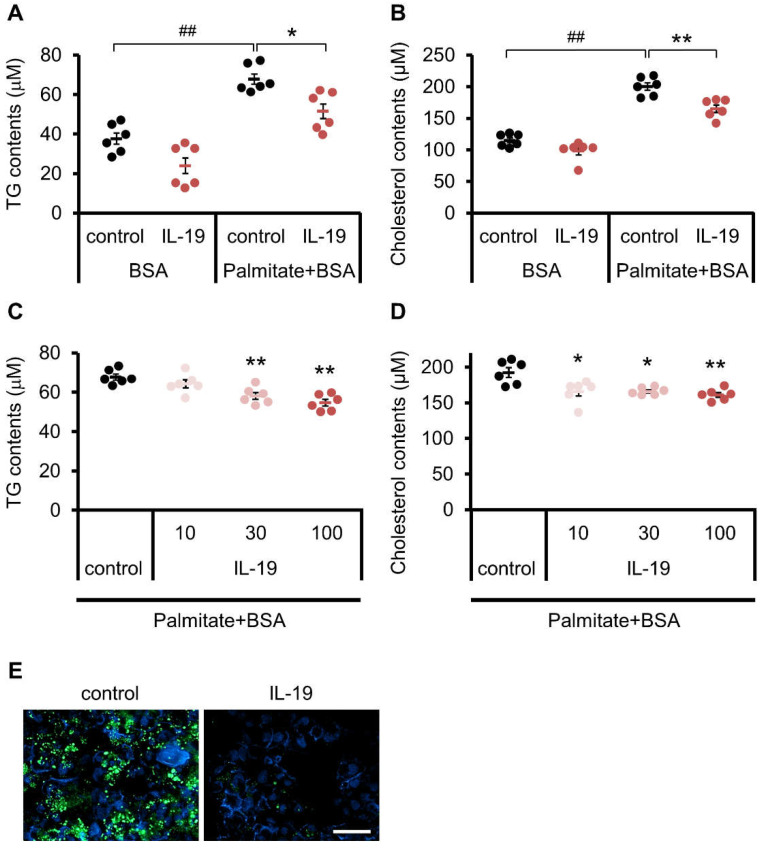
The changes in TG and cholesterol contents. (**A**,**B**,**E**) HepG2 cells were treated with 200 μM palmitate-BSA and 100 ng/mL IL-19 for 48 h. (**C**,**D**) HepG2 cells were treated with 200 μM palmitate-BSA and IL-19 for 48 h. (**A**,**C**) The TG levels were measured (*n* = 6). (**B**,**D**) The cholesterol levels were measured (*n* = 6). (**E**) Intracellular TG were visualized by BODIPY (493/503). Representative images are shown (*n* = 3). Scale bar represents 50 μm. ^##^ *p* < 0.01 vs. BSA. * *p* < 0.05, ** *p* < 0.01 vs. control.

We have shown that liver fibrosis is exacerbated in association with IL-19 gene deletion. In order to clarify the mechanism of action of IL-19, we conducted further experiments using in vitro steatosis models in HepG2 cells. Treatment of HepG2 cells with palmitate resulted in an accumulation of TG and cholesterol (Figure 5). Treatment with IL-19 abolished the accumulation of TG (Figure 5E). When quantified, TG content was significantly increased by palmitate-BSA (Palmitate), and the palmitate-treated increase was significantly decreased by IL-19 treatment in a concentration-dependent manner (Figure 5A,C). As well as the TG content, cholesterol content was significantly increased by palmitate and palmitate-treated increase was significantly decreased by IL-19 treatment in a concentration-dependent manner (Figure 5B,D).

**Figure 6:**
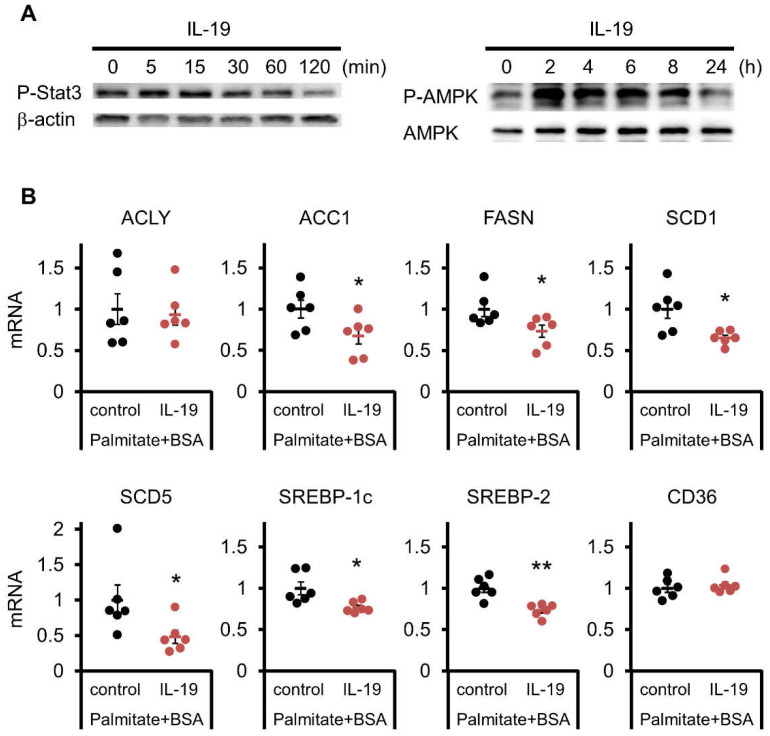
Signaling factors that respond to IL-19. (**A**) HepG2 cells were treated with 100 ng/mL IL-19. Total cell lysates were prepared and blotted with antibodies against the phosphorylated form of STAT3 and phosphorylated and non-phosphorylated forms of AMPK. Representative images are shown (*n* = 4). (**B**) HepG2 cells were treated with 200 μM palmitate-BSA and 100 ng/mL IL-19 for 48 h. The mRNA expression levels of each factor were evaluated by QPCR (*n* = 6). * *p* < 0.05, ** *p* < 0.01 vs. control.

We have shown that IL-19 seems to be involved in fat digestion. Next, the changes in factors and enzymes involved in fatty acid synthesis and other processes were analyzed by Western blotting and QPCR. IL-19 activates STAT3 phosphorylation among the Jak-STAT pathway [7]. We found that phosphorylation of STAT3 was enhanced by IL-19 treatment in HepG2 cells (Figure 6A, left). We also tested that this phosphorylation of STAT3 is cancelled by the JAK1/2 inhibitor Ruxolitinib (data not shown). Interestingly, we found that AMPK phosphorylation was enhanced by IL-19 treatment (Figure 6A, right). By QPCR, IL-19 treatment resulted in significant suppression of acetyl-CoA carboxylase (ACC) 1, fatty acid synthase (FASN), stearoyl-CoA desaturase (SCD) 1 and 5, sterol regulatory element-binding protein (SREBP)-1c and 2 (Figure 6B). In contrast, there were no clear changes in ATP citrate lyase (ACLY) and CD36.

**Figure 7:**
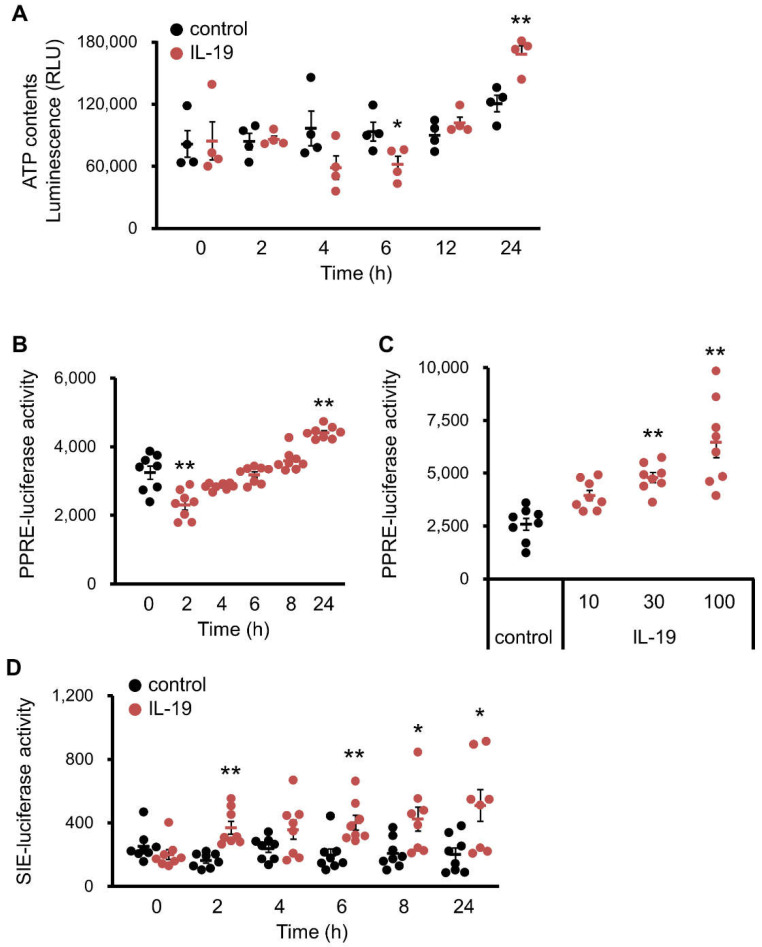
The changes in ATP content and the luciferase activities of PPRE and SIE. (**A**) HepG2 cells were treated with 200 μM palmitate and 100 ng/mL IL-19. The ATP content was measured (*n* = 4). * *p* < 0.05, ** *p* < 0.01 vs. control. (**B**) HepG2 cells with expression of PPRE-luciferase were treated with 100 ng/mL IL-19. The luciferase activity was measured (*n* = 8). ** *p* < 0.01 vs. 0 h. (**C**) HepG2 cells with expression of PPRE-luciferase were treated with IL-19 for 24 h. The luciferase activity was measured (*n* = 8). ** *p* < 0.01 vs. control. (**D**) HepG2 cells with expression of SIE-luciferase were treated with 100 ng/mL IL-19. The luciferase activity was measured (*n* = 8). * *p* < 0.05, ** *p* < 0.01 vs. control.

Since IL-19 was found to affect fatty acid synthesis, we therefore examined the effect of IL-19 on ATP production by β-oxidation. As shown in Figure 7A, IL-19 showed a significant increase in ATP production at 24 h in HepG2 cells, although suppression was observed at 6 h. We then proceeded to analyze the activity of PPARα, which are important in lipid metabolism in the liver [26]. PPRE-luciferase activity in HepG2 cells was increased at 24 h by IL-19 treatment, although suppression was observed at 2 h (Figure 7B). In addition, IL-19 significantly increased PPRE-luciferase activity in a concentration-dependent manner (Figure 7C). Thus, IL-19 can regulate β-oxidation via PPAR in HepG2 cells. Moreover, we proceeded to analyze the activity of SIE, which is a site of transcriptional regulation via STAT3. SIE-luciferase activity in HepG2 cells was persistently elevated from 2 to 24 h after IL-19 treatment (Figure 7D).

### 3.7. Effect of IL-19 on the Response in Hepatocyte

**Figure 8:**
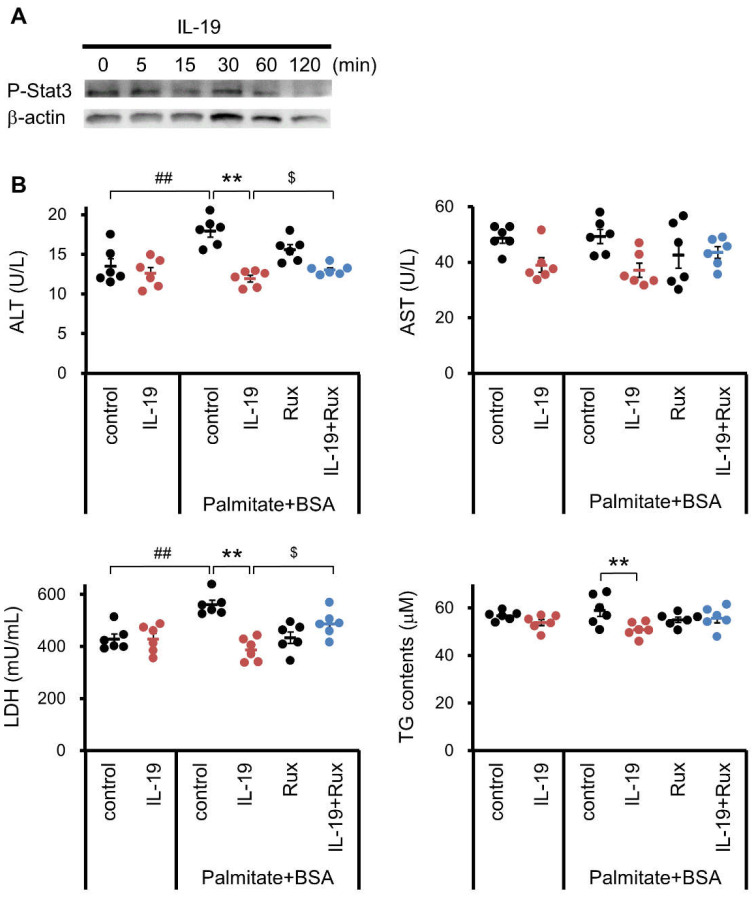
Various responses of primary hepatocytes that respond to IL-19. (**A**) Primary hepatocytes were treated with 100 ng/mL IL-19. Total cell lysates were prepared and blotted with antibody against the phosphorylated form of STAT3. Representative images are shown (*n* = 4). (**B**) Primary hepatocytes were treated with 200 μM palmitate-BSA, 100 ng/mL IL-19, and 2 μM ruxolitinib for 48 h. The ALT and AST levels were measured (*n* = 6). The LDH levels were measured (*n* = 6). The TG levels were measured using (*n* = 6). ^##^ *p* < 0.01 vs. BSA. ** *p* < 0.01 vs. control. ^$^ *p* < 0.05 vs. IL-19.

In addition to the analysis in HepG2 cells, we used the primary hepatocytes to analyze some responses caused by IL-19 treatment. Primary hepatocytes were treated with palmitate and analyzed in a similar experiment as HepG2 cells. Similar to HepG2 cells, hepatocytes showed increased phosphorylation of STAT3 upon IL-19 treatment (Figure 8A). In addition, IL-19 significantly suppressed the increase of ALT and LDH by palmitate in hepatocytes. The IL-19-treated decreases of ALT and LDH were cancelled by the JAK1/2 inhibitor Ruxolitinib (Figure 8B). IL-19 significantly suppressed the TG content in hepatocytes (Figure 8B).

## 4. Discussion

The diet used in this study was a custom-made diet with cholesterol added to a commercial diet (#A06071302), and this is the first report of its use in mice. After 9 weeks of feeding, WT mice showed increased liver weight, increased ALT, increased pro-inflammatory cytokines, and marked steatosis, while fibrosis was weak. These results indicate that the WT mice phenotype is in NAFL or the early stage of NASH. Importantly, IL-19 KO mice showed marked fibrosis in addition to increased liver weight, increased ALT, increased pro-inflammatory cytokines, and marked steatosis. On the other hand, AST did not show any significant difference. Due to the high absolute amount of AST, AST levels rise rapidly in acute hepatitis, including rapid necrosis of hepatocytes. As AST has a shorter half-life than ALT, ALT levels are higher in inactive chronic hepatitis. Therefore, since the present analysis is based on a chronic model, it is reasonable to suggest that there was a significant increase in ALT and no difference in AST. A further detailed comparison of the results of WT and IL-19 KO mice showed that liver weight and steatosis were milder in IL-19 KO mice than in WT mice, and increases in ALT and pro-inflammatory cytokines were worse in IL-19 KO mice than in WT mice. Generally, inflammatory cytokines and fibrosis increase as progression from NAFL to NASH in NAFLD in humans, but the degree of steatosis decreases and liver weight is reduced [27]. Thus, these results indicate that the IL-19 KO mice phenotype has progressed to NASH.

This custom-made diet first caused steatosis due to fat accumulation, followed by fibrosis. The action of IL-19 may control steatosis formation, fibrosis formation, or both. Although there are limitations to evaluating only one endpoint, the data of CD68-positive macrophages around oily cells are interesting. Since CD68-positive macrophages accumulated around fat droplets, IL-19 may be involved in metabolic abnormalities of hepatocytes. Since hepatocytes used in vitro were apparent to non-IL-19 producing cells, our results indicate that the cells producing IL-19 are Kupffer cells, the cells on which IL-19 produced in Kupffer cells acts are hepatocytes, and the result of the action is suppression of lipid metabolism. In vitro models of steatosis using HepG2 cells revealed novel effects of IL-19. IL-19 inhibited ACC1 and FASN, indicating that IL-19 inhibits fatty acid synthesis in the cells [28]. As a result, IL-19 contributes to a shift in lipid metabolism from fatty acid synthesis to promotion of β-oxidation. Furthermore, IL-19 suppressed SCD1 and SCD5, which means that saturated fatty acids do not become unsaturated fatty acids and the synthesis of triglycerides is suppressed [29]. These data suggest that IL-19 enhances β-oxidation and the digestion of fat. In addition, IL-19 inhibited cholesterol contents and suppressed SREBP-1c and SREBP-2 that stimulate HMG-CoA reductase, followed by cholesterol synthesis [30]. In addition, SREBPs promote the regulation of ACLY, ACC1, FASN, and SCD1, which are involved in fatty acid synthesis [31]. The data supporting this possibility is that IL-19 increased ATP production. The liver contains PPARα, which are important factors involved in fat metabolism. One of the key findings of this new study is that IL-19 increased PPRE-luciferase activity. When PPARα is stimulated, fatty acids are β-oxidized and become an energy source, inhibiting the synthesis of TG and VLDL [32]. We elucidated the signaling pathways that lead to the suppression of fatty acid synthesis and TG levels. First, IL-19 treatment resulted in the phosphorylation of STAT3 and increased SIE-luciferase activity. Importantly, IL-19 treatment resulted in the phosphorylation of AMPK. In the liver, AMPK inhibits fatty acid synthesis by suppressing ACC1 and SREBPs and produces energy by promoting β-oxidation [33]. Among factors and enzymes involved in the fatty acid synthesis measured, no significant change was observed for ACLY and CD36. A possible reason is that AMPK inhibits ACC but not ALCY. Although SREBPs regulated ACLY and ACC, the effect of IL-19 is stronger on ACC because AMPK is upstream of SREBPs. CD36 is a transporter of fatty acids. There was no change in CD36, suggesting that IL-19 does not affect the uptake of fatty acids. In summary, IL-19 inhibited lipogenesis in the liver.

In the present study, we found that IL-19 KO mice progressed from NAFLD to NASH. Therefore, IL-19 may play an important role in inhibiting the development of NAFLD. The data supporting this possibility is supported by in vitro results. The pathophysiology of NASH is very complex and many cell types within the liver are involved at multiple levels [34]. In whole liver, the factors measured by QPCR were IL-6, TNF-α, and TGF-β. We now focus on TGF-β. TGF-β is a widely known promoter of fibrosis in the liver [35]. Hepatic stellate cells are a key player in the development of fibrosis and are a major producer of extracellular matrices, such as collagen [36]. Hepatic stellate cells express TGF-β receptors and collagen productions are activated by TGF-β [37]. Macrophages in the liver can be divided into two types: resident Kupffer cells and bone marrow-derived macrophages, which infiltrate in response to inflammation or injury. These resident Kupffer cells and bone marrow-derived macrophages produce TGF-β, which directly activates the hepatic stellate cells [38]. In addition, hepatocytes also produce TGF-β in small amounts. Therefore, it is suggested that either or all of Kupffer cells, bone marrow-derived macrophages, or hepatocytes may have increased TGF-β productions in response to IL-19 gene deletion. We next turn our attention to IL-6 and TNF-α. For IL-6 and TNF-α, Kupffer cells and bone marrow-derived macrophages are the major producers. The produced IL-6 and TNF-α first act on hepatocytes to promote TGF-β productions and also directly activate the hepatic stellate cells [39]. With the same considerations as TGF-β, it is suggested that Kupffer cells and bone marrow-derived macrophages have increased IL-6 and TNF-α productions in response to IL-19 gene deletion. In our previous report, bone marrow-derived macrophages from IL-19 KO mice produced significantly higher levels of IL-6 and TNF-α than WT mice after stimulation with lipopolysaccharide [4]. Therefore, it seems likely that IL-19 deficiency also induced an increase in pro-inflammatory cytokines in Kupffer cells and macrophages in the liver tissue. In this study, we clarified the role of IL-19 on lipid metabolism. Future analysis of the effect of IL-19 on hepatic stellate cells will be necessary to focus on fibrosis formation.

## 5. Conclusions

Despite the extensive role of IL-19 in various organs of the body, its role in liver diseases, especially in chronic liver diseases such as fatty liver and NASH, is completely unexplored. In conclusion, this is the first report that IL-19 KO mice exacerbated NASH progression, IL-19 inhibited steatosis and fibrosis by directly regulating liver metabolism, and IL-19 plays an important role in liver disease.

## Data Availability

Not applicable.

## Supplementary Materials

The following are available online at https://www.mdpi.com/article/10.3390/cells10123513/s1, Table S1: List of specific primers used.

## Abbreviations

ACC	acetyl-CoA carboxylase
ACLY	ATP citrate lyase
ALT	alanine aminotransferase
AST	aspartate aminotransferase
CDAHFD	60% high fat, 0.1% methionine, and 2% cholesterol without choline
FASN	fatty acid synthase
H&E	hematoxylin and eosin
IL	interleukin
KO	gene-deficient
NAFLD	nonalcoholic fatty liver disease
NAFL	nonalcoholic fatty liver
NASH	nonalcoholic steatohepatitis
PPAR	peroxisome proliferator-activated receptor
PPRE	PPAR-response element
QPCR	quantitative Real-Time PCR
SCD	stearoyl-CoA desaturase
SD	standard diet
SIE	sis-inducible element
SREBP	sterol regulatory element-binding protein
TG	triglyceride
WT	wild-type
