# Supplementary material for: IL-19 Contributes to the Development of Nonalcoholic Steatohepatitis by Altering Lipid Metabolism
Source: Cells. 2021 Dec 13;10(12):3513. doi: 10.3390/cells10123513 (PMC8699936; doi:10.3390/cells10123513)
Supplement: Supplementary file 1 [file cells-10-03513-s001.zip › cells-1491937-supplementary.pdf]

Supplementary Table S1. List of specific primers used

gene	forward	reverse	accession number	product length (bp)
Il-19	CTCCTGGGCATGACGTTGATT	GCATGGCTCTCTTGATCTCGT	NM_001009940.2	118
Il-6	CTGCAAGAGACTTCCATCCAG	AGTGGTATAGACAGGTCTGTTGG	NM_031168.2	131
TNF-α	CATCTTCTCAAAATTCGAGTGACAA	TGGGAGTAGACAAGGTACAACCC	NM_013693.3	175
TGF-β	TACAGGGCTTTCGATTCAGC	CGCACACAGCAGTTCTTCTC	NM_011577.2	247
ACLY	TGCTCGATTATGCACTGGAAGT	ATGAACCCCATACTCCTTCCCAG	NM_001096	202
ACC1	GCTCCTTGTCACCTGCTTCT	CAAGGCCAAGCCATCCTGTA	NM_198834	80
FASN	TCGTGGGCTACAGCATGGT	GCCCTCTGAAGTCGAAGAAGAA	NM_004104.5	78
SCD1	TCTAGCTCCTATACCACCACCA	TCGTCTCCAACTTATCTCCTCC	NM_005063.5	82
SCD5	ATGTCGTCCTGATGAGCTTG	CAGGAGGAAGCAGAAGTAGG	NM_001037582	107
SREBP-1c	AAACTCAAGCAGGAGAACCTAAGTCT	GTCAGTGTGTCCTCCACCTCAGT	NM_001005291	143
SREBP-2	CAGCAGCCTTTGATATACCAGAATG	AGGATGTCACCAGGCTTTGGAC	NM_004599	79
CD36	TCTTTCCTGCAGCCCAATG	AGCCTCTGTTCCAACTGATAGTGA	NM_000072	60
GAPDH	ACCCACTCCTCCACCTTTG	CTCTTGTGCTCTTGCTGGG	NM_001256799.3	178
HPRT	GTTGGATACAGGCCAGACTTTGTTG	GAGGGTAGGCTGGCCTATAGGCT	NM_013556.2	352
